# "Arming" the chimeric oncolytic adenovirus enadenotucirev to deliver checkpoint inhibitors and other therapeutics directly to tumours

**DOI:** 10.1186/2051-1426-2-S3-P46

**Published:** 2014-11-06

**Authors:** Brian R Champion, Prithvi Kodialbail, Sam Illingworth, Nalini Rasiah, Dan Cochrane, John Beadle, Kerry Fisher, Alice CN Brown

**Affiliations:** 1 PsiOxus Therapeutics Ltd, Abingdon, United Kingdom

## 

Enadenotucirev (EnAd; formerly called ColoAd1) is a potent, chimeric Ad11p/Ad3 adenovirus active against a range of epithelial cancer cells, with a shorter time-to-lysis than either wild type Ad11p, Ad3 or Ad5. In normal cells, EnAd is attenuated and shows little or no activity by either cytotoxicity or by qPCR. In vivo, EnAd shows efficacy in a range of xenograft human tumour models following intra-tumoural, intravenous and intra-peritoneal injection [1] and is currently being evaluated clinically for treatment of several different epithelial cancers. Data from an ongoing clinical mechanism of action study have shown that i.v. dosed EnAd infects and replicates in tumour cells, producing significant amounts of viral protein (hexon), indicating that transgene encoded proteins will also be made in significant amounts by tumours following i.v. delivery of an armed EnAd virus. To develop 'armed' variants for delivery of therapeutic agents that enhance EnAd's anti-tumour activity, we have developed a novel, efficient cloning system for rapid generation of modified viruses that can be dosed systemically to deliver immunomodulatory antibodies into tumours. We chose to first encode anti-VEGF antibodies since, unlike immunomodulators, they could be readily evaluated in vivo in immunodeficient mouse human tumour xenograft models. We have successfully produced EnAd variants encoding full-length (NG-135) and ScFv (NG-76) forms of anti-human VEGF antibodies which have similar virus activity profiles to EnAd in cancer cell lines in vitro (virus replication, gene expression and oncolytic action), but also express and release the respective anti-VEGF antibody forms into the culture supernatant. Using either HCT-116 or DLD human colon carcinoma xenograft models we have shown that the virus infection profile following intra-tumoural injection is similar to the parental EnAd virus (virus replication and hexon gene expression). Anti-VEGF antibody expression by these tumours could be detected in the tissue as both mRNA and functional antibody. Antibodies were detectable early (within 3 days of infection) and expression was sustained over several weeks. Furthermore, low levels of anti-VEGF antibody were detectable in the blood. Production and evaluation of viruses similarly expressing checkpoint inhibitor antibodies is now in progress, together with evaluation of anti-VEGF armed oncolytic viruses for their impact on the growth and microenvironment of tumour xenografts.
